# Thermostatic properties of nitrate molten salts and their solar and eutectic mixtures

**DOI:** 10.1038/s41598-018-28641-1

**Published:** 2018-07-11

**Authors:** B. D’Aguanno, M. Karthik, A. N. Grace, A. Floris

**Affiliations:** 1 Centre for Nanotechnology Research, VIT University, Vellore, 632014 Tamil Nadu India; 2 Centre for Nanomaterials, International Advanced Research Centre for Powder Metallurgy and New Materials (ARCI), Balapur, Hyderabad, 500005 India; 3 School of Mathematics and Physics, University of Lincoln, Brayford Pool, LN6 7TS Lincoln, United Kingdom

## Abstract

Nitrate molten salts are extensively used for sensible heat storage in Concentrated Solar Power (CSP) plants and thermal energy storage (TES) systems. They are the most promising materials for latent heat storage applications. By combining classical molecular dynamics and differential scanning calorimetry experiments, we present a systematic study of all thermostatic, high temperature properties of pure KNO_3_ and NaNO_3_ salts and their eutectic and ”solar salt” mixtures, technologically relevant. We first study, in solid and liquid regimes, their mass densities, enthalpies, thermal expansion coefficients and isothermal compressibilities. We then analyze the *c*_*P*_ and *c*_*V*_ specific heats of the pure salts and of the liquid phase of the mixtures. Our theoretical results allow to resolve a long-standing experimental uncertainty about the *c*_*P*_(*T*) thermal behaviour of these systems. In particular, they revisit empirical laws on the *c*_*P*_(*T*) behaviour, extensively used at industrial level in the design of TES components employing the ”solar salt” as main storage material. Our findings, numerically precise and internally consistent, can be used as a reference for the development of innovative nanomaterials based on nitrate molten salts, crucial in technologies as CSP, waste heat recovery, and advanced adiabatic compressed air energy storage.

## Introduction

Molten salts are the most used materials for thermal energy storage at high temperature. This is due to several physical properties that they exhibit, which are important in industrial applications related to energy. The first factor affecting the performance of a thermal energy storage (TES) system is the thermal stability of the materials used to store heat, i.e. the temperature interval where they are liquid. The thermal stability of nitrate molten salts (MNO_3_, M = alkali metal), allows the heat to be stored between ≈520 K and ≈890 K, an extended range of very high temperatures. This interval is typical for pure alkali nitrates (LiNO_3_, NaNO_3_, KNO_3_), while their mixtures show a lower freezing point (e.g. for the eutectic NaNO_3_-KNO_3_ mixture this is 495 K). This even larger thermal stability range fits the requirements of Concentrated Solar Power (CSP) plants which, as a consequence, use nitrate molten mixtures as a heat storage medium. By 2030, it is estimated a usage of ≈1.8 × 10^9^ tons of nitrate mixtures in CSP plants^1^.

Despite this extensive use of molten salts in thermal energy management, many basic and challenging issues about these materials are still unresolved, both at the experimental and theoretical level. One crucial issue is the temperature dependence of *c*_*P*_(*T*) in their liquid phase, especially concerning nitrate molten mixtures. Experimentally, different dependencies have been found, from increasing, to constant, to decreasing with *T*^2–7^. For this reason, round robin tests have been launched within the scientific and technological communities^8^.

A technologically relevant nitrate mixture is the so called “solar salt”, which has a weight fraction composition given by ${x}_{NaN{O}_{3}}=0.6$ and ${x}_{KN{O}_{3}}=1-{x}_{NaN{O}_{3}}=0.4$. Industrially, for this mixture the most used empirical equation for the mass density *ρ*(*T*), at atmospheric pressure and within 530–890 K, is the function *ρ*(*T*) = *α* − *β T*, where *α* = 2.09 and *β* = 0.000636^9^. The empirical relation used for the specific heat is the *T-increasing* function *c*_*P*_(*T*) = *γ* + *δ T* where *γ* = 1.443 and *δ* = 0.000172^9^ (the units of *T*, *ρ*(*T*) and *c*_*P*_(*T*) are K, g cm^−3^ and Jg^−1^ K^−1^, respectively). This last equation is the result of many experimental measurements and statistical analyses all based on the use of Differential Scanning Calorimetry (DSC)^10,11^. However, an increasing *c*_*P*_(*T*) behaviour is more typical of weakly interacting liquids^12^, while nitrates belong to the class of strongly interacting ones.

In this rather complex experimental scenario, systematic theoretical results about thermostatic properties of molten salts and their mixtures are largely missing, while results for enthalpy *H*(*T*) and *c*_*P*_(*T*) of pure salts have been reported in ref.^13^.

As mentioned, empirical equations represent a key factor in establishing TES performances and have been crucial in the design of state-of-the-art CSP plants built recently^14^. Thus, revisiting them theoretically has a potentially high technological impact. Here, the validity of the mentioned *c*_*p*_ relation will be investigated in detail and critically reconsidered.

Clearly, improvements in the performances of storage systems, connected to the energy production and reduction of electricity costs (e.g., of CSP plants), heavily rely on the maximum optimization of the thermodynamic properties of well known and new salt-based mixtures^15–19^.

In this work, we perform an extensive theoretical analysis, based on classical molecular dynamics (MD), of the temperature behavior of the thermostatic properties of NaNO_3_, KNO_3_ and their mixtures, with emphasis on the thermal behaviour of the specific heats *c*_*P*_(*T*) and *c*_*V*_(*T*), technologically relevant especially for the mixtures. The results are first compared with our new DSC experiments and with previous measurements. Then, they are interpreted by establishing a link between the solid-state approach to collective vibrational modes in liquids^20–23^, and the more standard gas-like approach^24,25^.

Our analysis is devoted to (*i*) provide a theoretical characterization, both in the solid and liquid phases, of the thermostatic properties of nitrate molten salts; (*ii*) resolve the issues related to the *c*_*P*_(*T*) thermal behavior in the liquid phase of relevant salt mixtures; (*iii*) validate the MD model^13^ accuracy, for further studies about, e.g., nanofluids where the suspending medium are the nitrate salts studied here. The first two points are relevant for all the applications in the CSP technology and more generally in the field of renewable energy; the third, to develop an accurate theoretical framework able to study the thermodynamic properties of salt-based fluids for thermal energy storage^15–19^. Our calculations are supported by a self-consistency check on the precision of our numerical methodology, only possible thanks to the fact that *all* relevant thermostatic properties were investigated.

## Results

We will first investigate the thermostatic properties of pure potassium and sodium nitrates, in their solid and liquid regimes. Next, we will analyze the eutectic and “solar” mixtures with the ultimate goal to characterize the thermal behaviour of the specific heats in the liquid phases. All properties and methods to calculate them are described in the Methods Sections (MS).

### Potassium Nitrate KNO_3_

#### Melting temperature

In order to study via MD the KNO_3_ properties in a large temperature regime including various phases, the starting point is to locate the solid-to-liquid transition temperature *T*_*M*_, at *P* = 1 atm. We find ${T}_{M}^{MD}=592.5\pm 2.5\,{\rm{K}}$, which is very close to the experimental value, ${T}_{M}^{exp}=607$ K. This excellent agreement gives a first indication of the accuracy of the inter-atomic potentials used^13^.

Moreover, it is also the result of a MD procedure to locate *T*_*M*_, based on the temporal evolution of a two-phase system, as described in MS 1.4. The melting point is also reported in ref.^13^, where a different value (*T*_*M*_ = 513 K ± 17 K) was obtained via a thermodynamic integration-based method.

**Figure 1:**
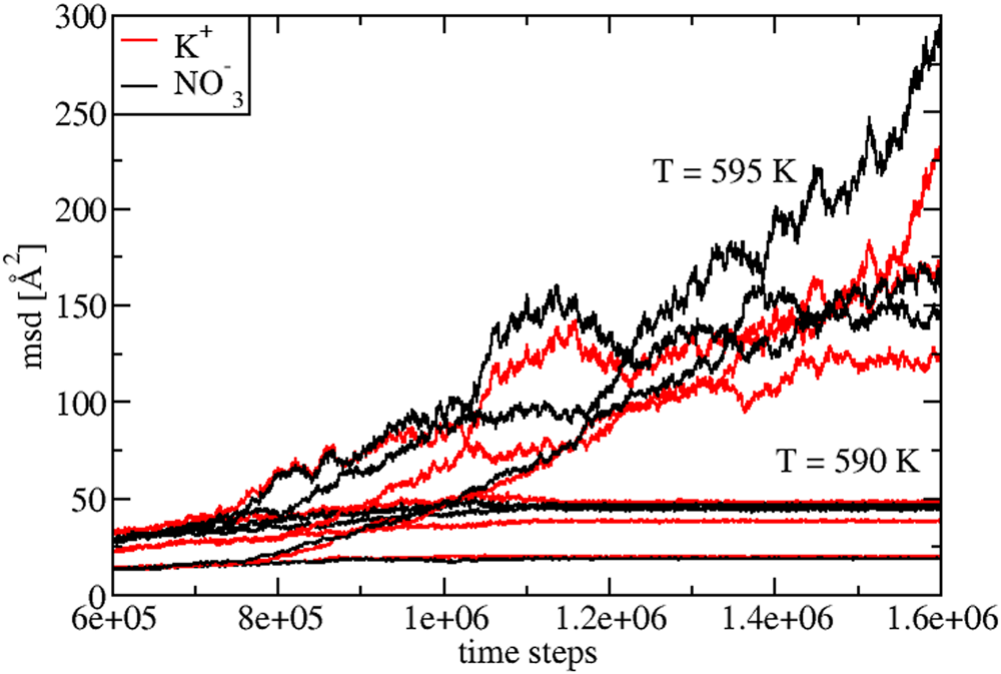
Mean squared displacement (*msd*) of K^+^ and ${{\rm{NO}}}_{3}^{-}$ ions, as a function of the molecular dynamics (MD) time steps. Data are shown for two temperatures, *T* = 590 K and *T* = 595 K. They are, respectively, immediately below and above the calculated melting temperature ${T}_{M}^{MD}$ = 592.5 K. Three cartesian components for each *T* are shown. At *T* = 590 K a typical solid-like constant behavior is evident, while at *T* = 595 K the plot exhibits a completely different, liquid-like behavior.

By following our procedure, in Fig. 1 we show the evolution of the three Cartesian components of the mean squared displacement (*msd*) of K^+^ and $${{\rm{NO}}}_{3}^{-}$$ at *T* = 590 K and *T* = 595 K. At *T* = 590 K ($$ < {T}_{M}^{MD}$$), the *msd* shows a typical solid-like constant behavior, while at *T* = 595 K ($$ > {T}_{M}^{MD}$$), a typical liquid-like behavior.

#### Mass density and thermal expansion coefficient

Experimentally, solid KNO_3_ shows three polymorphic forms at *P* = 1 atm^26^: a stable form at 299 K, denoted *α*-KNO_3_; a stable phase generated by heating at *T* = 403 K, *β*-KNO_3_; and a third, different metastable phase *γ*-KNO_3_ obtained by cooling down the system from high temperature, resulting from an alternative kinetic path.

**Figure 2:**
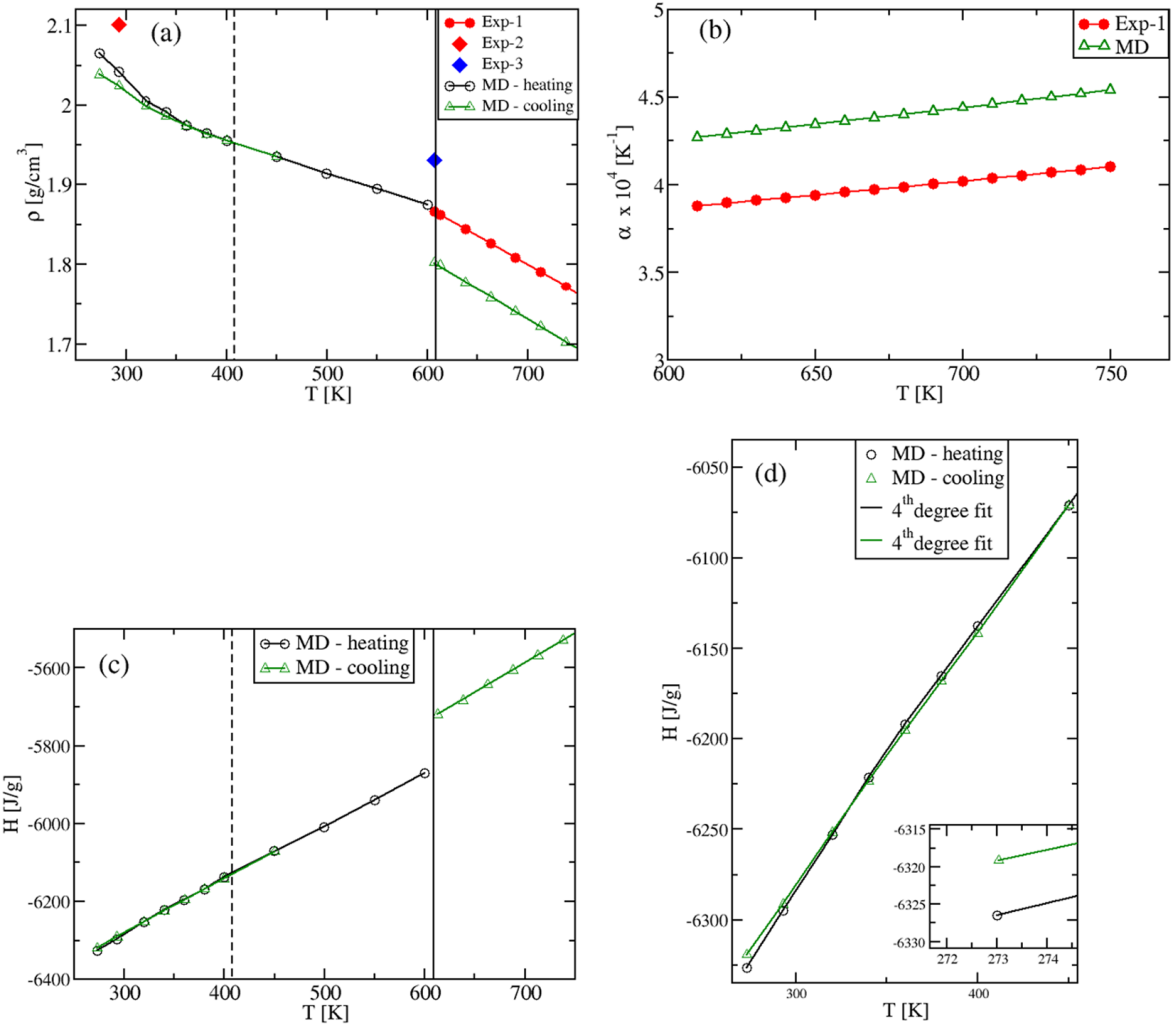
(**a**) Density *ρ*(*T*), (**b**) thermal expansion coefficient *α*(*T*) and (**c,d**) enthalpy *H*(*T*) of KNO_3_. *ρ*(*T*) and *H*(*T*) are calculated within *T* = 273–750 K at *P* = 1 atm; *α*(*T*) is computed in the liquid regime. (**d**) is a zoom of (**c**) within 273–450 K. Black and green plots are MD results calculated in this work. Red and blue full symbols are experimental results (Exp-1: ref.^36^; Exp-2: ref.^49^; Exp-3: ref.^27^). Vertical dashed lines correspond to the *α*-KNO_3_ to *β*-KNO_3_ transition (at the experimental ${T}_{S-S}^{exp}=403$ K), while full vertical lines indicate the melting transition (at ${T}_{M}^{exp}=607\,{\rm{K}}$). In (**a**), the splitting of the two green and black plots in the region *T* ≈ [273, 345] K is due to the KNO_3_ polymorphism, where two phases are obtained depending on the kinetic path followed by the system. In (**c**), we show the overall entalphy behavior in all investigated phases. The two different sets of data (black and green symbols) are obtained to heating up and cooling down the system. Both are best fitted with 4^*th*^ degree polynomials in the solid range, while in the liquid region we find a perfectly linear behavior. The inset in (**d**) shows that a lower enthalpy is associated to *α*-KNO_3_, which is more stable than *γ*-KNO_3_ at T = 273 K.

As we aim to characterize the specific heats in various phases, we preliminary analyze the density *ρ*(*T*) and the enthalpy *H*(*T*) temperature behavior. The results are presented in Fig. 2. Focusing first on the solid phases between 273 K and ≈600 K, we find that by heating up the *α*-KNO_3_ phase from *T* = 273 K, the calculated density shows a strongly non-linear behavior in the range *T* = [273,400] K, Fig. 2(a). This indicates the formation of a new phase, corresponding to *β*-KNO_3_. By cooling down the latter from *T* = 450 K, the density is once again non-linear, but the *γ*-KNO_3_ phase is obtained at room temperature.

In the liquid region, the density *ρ*(*T*) is, instead, a linear function with a MD slope that coincides with the experimental one^27^. In this regime, our values confirm quantitatively the ones reported previously^13^. The slope, normalized by *ρ*(*T*), gives the thermal expansion coefficient *α*(*T*), shown in Fig. 2(b). The difference between experiment and theory in *α*(*T*) reflects only the difference in the corresponding values of the density, which is within 5% in the entire *T*-range investigated (at *T* = 293 K, *ρ* = 2.023 g cm^−3^, while *ρ*^*exp*^ = 2.101 g cm^−3^; at *T* ≈ 600 K, *ρ* = 1.8 g cm^−3^, while *ρ*^*ex*^ = 1.867 g cm^−3^). A good agreement is also obtained for the volume change at the experimental melting point, *T*_*M*_ = 607 K. At ${T}_{M}^{MD}=592.5\,{\rm{K}}$, the MD value is ${\rm{\Delta }}{V}_{m}^{MD}/{V}_{S}^{MD}=4.03$%, while the experimental change is ${\rm{\Delta }}{V}_{m}^{exp}/{V}_{S}^{exp}=3.3$%^27^. Again, these density results confirm the ability of the inter-atomic potentials^13^ in describing the experimental findings.

#### Enthalpy

We next calculate the enthalpy *H*(*T*) in the whole temperature interval. In Fig. 2(c,d), a non-linear *T*- behavior corresponding to the density one is found in the solid range. The two MD plots in Fig. 2(c) are obtained by heating up or cooling down the system, respectively (black and green symbols). The values at *T* = 273 K show that the stable phase is the *α*-KNO_3_ (Fig. 2(d) inset), while the metastable *γ*-KNO_3_ phase should transform into the former with time^28^. In the liquid phase we find the important result that our *H*(*T*) data are perfectly *linear* with the temperature. Finally, by extrapolating the solid and liquid *H*(*T*) to *T*_*M*_, the computed value for the melting enthalpy is ${\rm{\Delta }}{H}_{M}^{MD}=130\,{\rm{J}}/{\rm{g}}$, which is to be compared with the experimental value of ${\rm{\Delta }}{H}_{M}^{\mathrm{Exp}}\approx 100\,{\rm{J}}/{\rm{g}}$^4^.

#### Specific Heats

**Figure 3:**
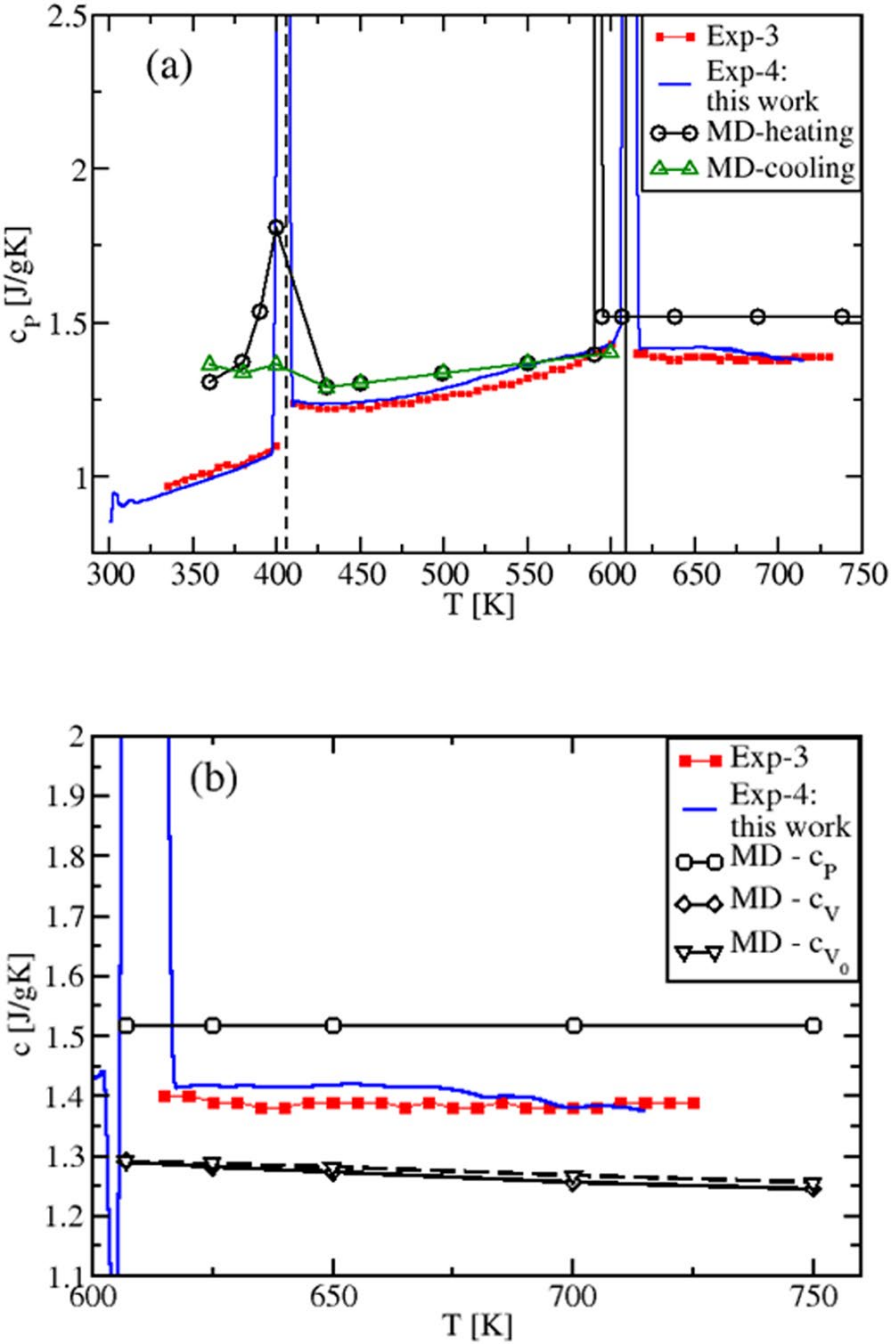
(**a**) Isobaric specific heat of KNO_3_, in the whole solid (at *P* = 1 atm) and liquid regimes investigated. In the solid phase, *c*_*P*_ is calculated both when heating (black) and cooling (green) the system. A transition peak is shown in correspondence of the solid-to-solid *α*-KNO_3_ → *β*-KNO_3_ phase transition (*T* ≈ 400 K)^26^ when heating, in excellent agreement with the experiments. (**b**) Specific heats *c*_*P*_, *c*_*V*_ and ${c}_{{V}_{0}}$ of KNO_3_ in the liquid *T*-range only. The simulated *c*_*P*_ is found to be *constant*, while *c*_*V*_ and ${c}_{{V}_{0}}$ are both decreasing with temperature. Exp-3: ref.^4^; Exp-4: this work.

By performing the derivative of the MD enthalpy plots (fitting polynomials for *H*(*T*) were used), we now show the obtained specific heats (Fig. 3), and compare them with our experimental results and existing experiments^4^. The isobaric *c*_*P*_(*T*) is presented in Fig. 3(a). Remarkably, due to the *H*(*T*) non-linearity in the solid regime, the calculated *c*_*P*_ shows a transition peak in correspondence of the solid-to-solid *α*-KNO_3_ → *β*-KNO_3_ transition (*T* ≈ 400 K)^26^ and, still in accordance with the experimental data, a slightly increasing behavior until the melting *T*_*M*_ is reached. Importantly, Fig. 3(b) shows that in the whole temperature range of liquid stability *c*_*P*_ has a *constant value*, ${c}_{P}^{MD}=1.518$ Jg^−1^ K^−1^, in disagreement with previous theoretical results^13^.

Figure 3(b) also shows the MD predicted *c*_*V*_(*T*) and ${c}_{{V}_{0}}(T)$ behaviors calculated, respectively, at constant (*V*) and at fixed volume (*V*_0_). The latter is chosen to be the volume immediately after the melting temperature^29^. As it is clear from the internal energy of the liquid state (Eq. (5), Sec. 1.2), the ${c}_{{V}_{0}}(T)$ decrease is purely due to the smoothing and broadening of the peaks in the pair distribution functions *g*_*ij*_(*r*), while in the *c*_*V*_(*T*) decrease there is the additional contribution from the variation of *V*(*T*, *P* = 1 atm), via the density *ρ*(*T*).

In the gas-like approach to liquids^24,25^, the *g*_*ij*_(*r*) smoothing and broadening correspond to less defined coordination shells around given ions, from which they can more easily escape or jump at increased temperature. In the solid-state approach of refs^20–23^, they correspond to a shorter time between two consecutive ion jumps, known as the Frenkel time *τ*_*F*_ (and to a larger frequency *ω*_*F*_). This results in a temperature decrease of the number of transverse oscillating modes, which has been identified as the main cause of the decrease of ${c}_{{V}_{0}}(T)$^20–23^.

Since *c*_*P*_(*T*) can be written as *c*_*P*_(*T*) = *c*_*V*_(*T*)(1 + *γαT*), where *γ* is the Grüneisen parameter and *α* is the coefficient of thermal expansion and since, typically, *γ* is almost *T*-independent^30^, it follows that *c*_*P*_(*T*) is the product of a temperature decreasing term, *c*_*V*_(*T*), and a temperature increasing term containing *αT*^2,20^. As a result, *c*_*P*_(*T*) must be *less*, or *not at all* sensitive to temperature changes, as we actually find. This behavior is also consistent with the experimental results found for molten alkali and alkali earth halides^31^ that, as KNO_3_, belong to the class of low viscosity ionic liquids. Previous *c*_*P*_(*T*) values^13^ differ from our results, since *c*_*P*_(*T*) was determined by considering, on the top of the MD interaction model, internal degrees of freedom contributions to the kinetic ideal term that are *T*-dependent^13^.

The overall agreement between experimental data and MD results in *T* = [400, 725] K is very good if compared to the accuracy found in the literature^13^, the difference being <8%. However, we note that the *c*_*P*_ experimental data are in between the *c*_*P*_ and *c*_*V*_ theoretical results and show a slight tendency to decrease and oscillate with increasing *T*. This could be a consequence of experimental conditions closer to constant *V* than to constant *P* (sealed and small sample holder used in DSC experiments).

#### Isothermal compressibility

**Figure 4:**
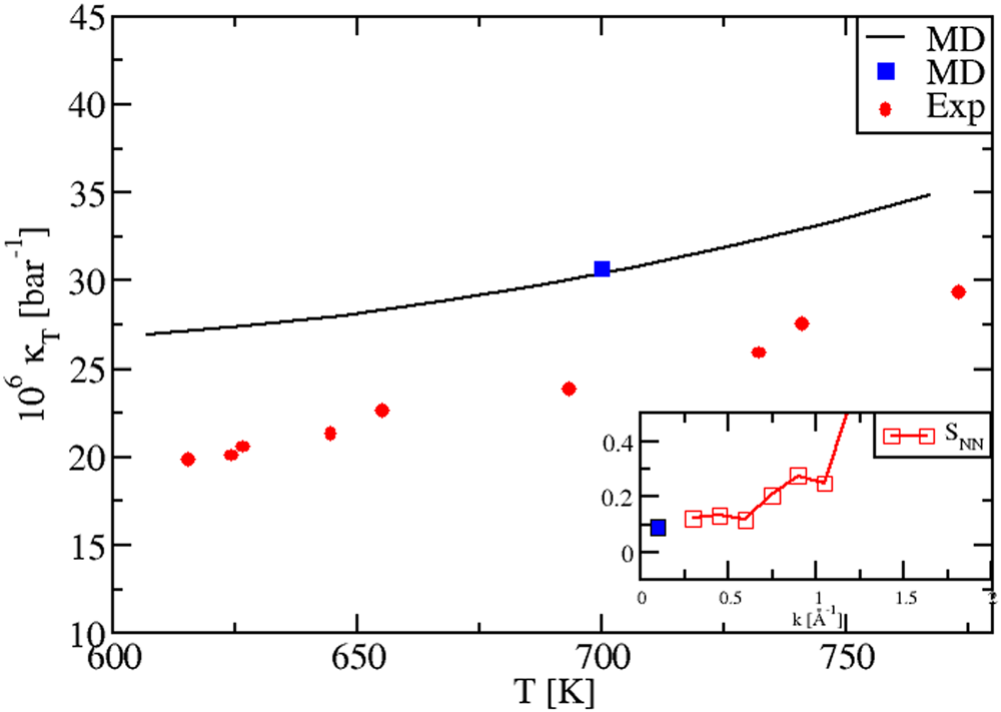
Isothermal compressibility *κ*_*T*_(*T*) of KNO_3_, calculated with *three* different methodologies. Exp: ref.^50^. The black plot is calculated from the relation *κ*_*T*_ = *α*^2^*T*/[*ρ*(*c*_*P*_ − *c*_*V*_)]. The blue dot at *T* = 700 K is computed via an independent procedure and using *κ*_*T*_ = −*V*^−1^(∂〈*V*〉/∂*P*)_*T*_. The blue dot is on top of the first plot, highlighting our internal consistency. In the inset, we report the *S*_*NN*_(*k*) Bhatia-Thornton structure factors^25,32,33^ calculated at *T* = 700 K. The blue dot corresponds to the previous one. We see that *S*_*NN*_(*k*) tends to the right limit for small wave vectors *k*, approaching a *κ*_*T*_ (blue dot) coming from a completely different calculation, confirming the validity of our numerical procedures.

Our calculations reproduce correctly also the temperature dependence of the isothermal *κ*_*T*_(*T*) in the liquid phase, Fig. 4. We evaluated *κ*_*T*_(*T*) by using *three* different procedures, fully illustrated in Methods Section 1.3. The black plot stems from the relation *κ*_*T*_ = *α*^2^*T*/[*ρ*(*c*_*P*_ − *c*_*V*_)]. The blue dot at *T* = 700 K is instead calculated via an alternative sequence of steps and the use of the equation *κ*_*T*_ = −*V*^−1^(∂〈*V*〉/∂*P*)_*T*_. Importantly, we can see that these data perfectly superpose. This represents a severe test to check our numerical precision and consistency, for *all* thermostatic quantities not directly obtainable as MD averages. We have a further confirmation of this consistency. After a suitable rescaling (MS, Eq. (9)) the blue dot value was reported in the inset of Fig. 4, together with the *S*_*NN*_(*k*) Bhatia-Thornton structure factors^25,32,33^, which we also calculated at *T* = 700 K. As it is well known^25,32,33^, we have the relation $\frac{1}{{\rho }_{N}{k}_{B}T}{\mathrm{lim}}_{k\to 0}{S}_{NN}(k)={\kappa }_{T}$. In the inset, we see that, indeed, our *S*_*NN*_(*k*) curve tends to the right limit for small wave vectors *k*, approaching the rescaled *κ*_*T*_ that comes from a separate calculation. Also in this case the internal consistency is evident.

### Sodium Nitrate NaNO_3_

We present now our results for NaNO_3_. To a large extent they exhibit similar features found in KNO_3_.

#### Melting temperature

The NaNO_3_ melting temperature is calculated to be ${T}_{M}^{MD}=594\,{\rm{K}}$, which is higher than the experimental value ${T}_{M}^{Exp}=581\,{\rm{K}}$^26^, but still in a reasonable agreement. ref.^13^ reports 591 ± 18 K.

#### Mass density and thermal expansion coefficient

**Figure 5:**
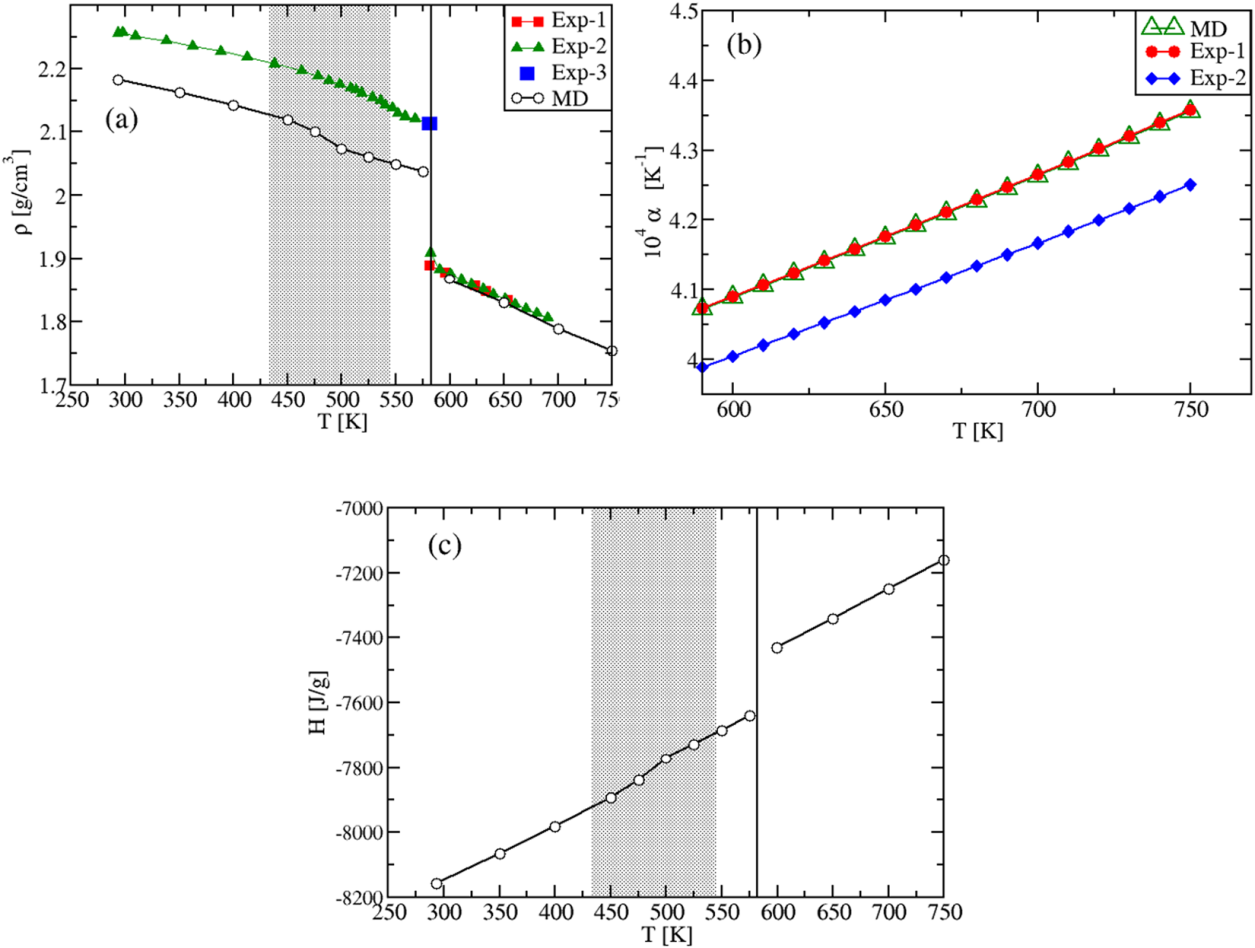
(**a**) Density *ρ*(*T*), (**b**) thermal expansion coefficient *α*(*T*) and (**c**) enthalpy *H*(*T*) of NaNO_3_. *ρ*(*T*) and *H*(*T*) are calculated in the full temperature range at *P* = 1 atm, *α*(*T*) in the liquid phase. Black and green plots with empty symbols are MD results calculated in this work. Full symbols are experimental results; Exp-1: ref.^36^; Exp-2: refs^2,51^; Exp-3: ref.^27^. In (**a**) and (**c**) vertical full lines indicate the experimental melting transition (${T}_{M}^{exp}\mathrm{=581}\,{\rm{K}}$), while shaded regions correspond to the second-order II-NaNO_3_ → I-NaNO_3_ phase transition (see text). The enthalpy MD results in the solid phase are fitted by a 4^*th*^ degree polynomial, while those in the liquid phase by a linear *T*-function. In (**b**), the MD data superpose with Exp-1, while the difference with Exp-2 is around 2%.

The mass density results are shown in Fig. 5(a). Also in this case, the experimental data in the solid range have a strong non-linear behavior, as in KNO_3_. In fact, at *T* = 433 K a second-order phase transition begins from the low temperature stable phase, denoted II-NaNO_3_, to the high temperature phase, I-NaNO_3_^34^. The transition is complete at *T* = 544 K^26^, and it is due to the activation of the rotational degrees of freedom of the nitrate groups^35^. In this temperature range, the theory-experiment discrepancy is found to be less than 4% at *T* = 293 K (*ρ*^*MD*^ = 2.181 g cm^−3^, while *ρ*^*exp*^ = 2.257 g cm^−3^), and the strong non-linearity is also well reproduced. Also in this case our values confirm quantitatively the ones reported previously^13^. In the liquid phase, both the *ρ*(*T*) linear behavior and the set of experimental data are perfectly reproduced. However, we need to consider that in the liquid range, the experimental data refer to systems where, intrinsically, both sodium nitrates and nitrites are present (see ref.^2^ and references therein), while our MD results refer to pure NaNO_3_. Still, although this level of precision might be quite accidental, the interaction potentials used show to be, once more, highly accurate. This is also true for the liquid branch of the thermal expansion coefficient, shown in Fig. 5(b), where we see that the theoretical and experimental^36^ results essentially superpose.

#### Enthalpy

The enthalpy *H*(*T*) is reported in Fig. 5(c). As for the density, the enthalpy is non-linear in the solid phase and linear in the liquid region. By extrapolating the solid and liquid enthalpies to the experimental ${T}_{M}^{exp}=581$ K, a value of ${\rm{\Delta }}{H}_{M}^{MD}=173$ J/g is obtained, in quantitative agreement with the experimental value, ${\rm{\Delta }}{H}_{M}^{Exp}\in 172-187$ J/g^2^.

#### Specific Heats

**Figure 6:**
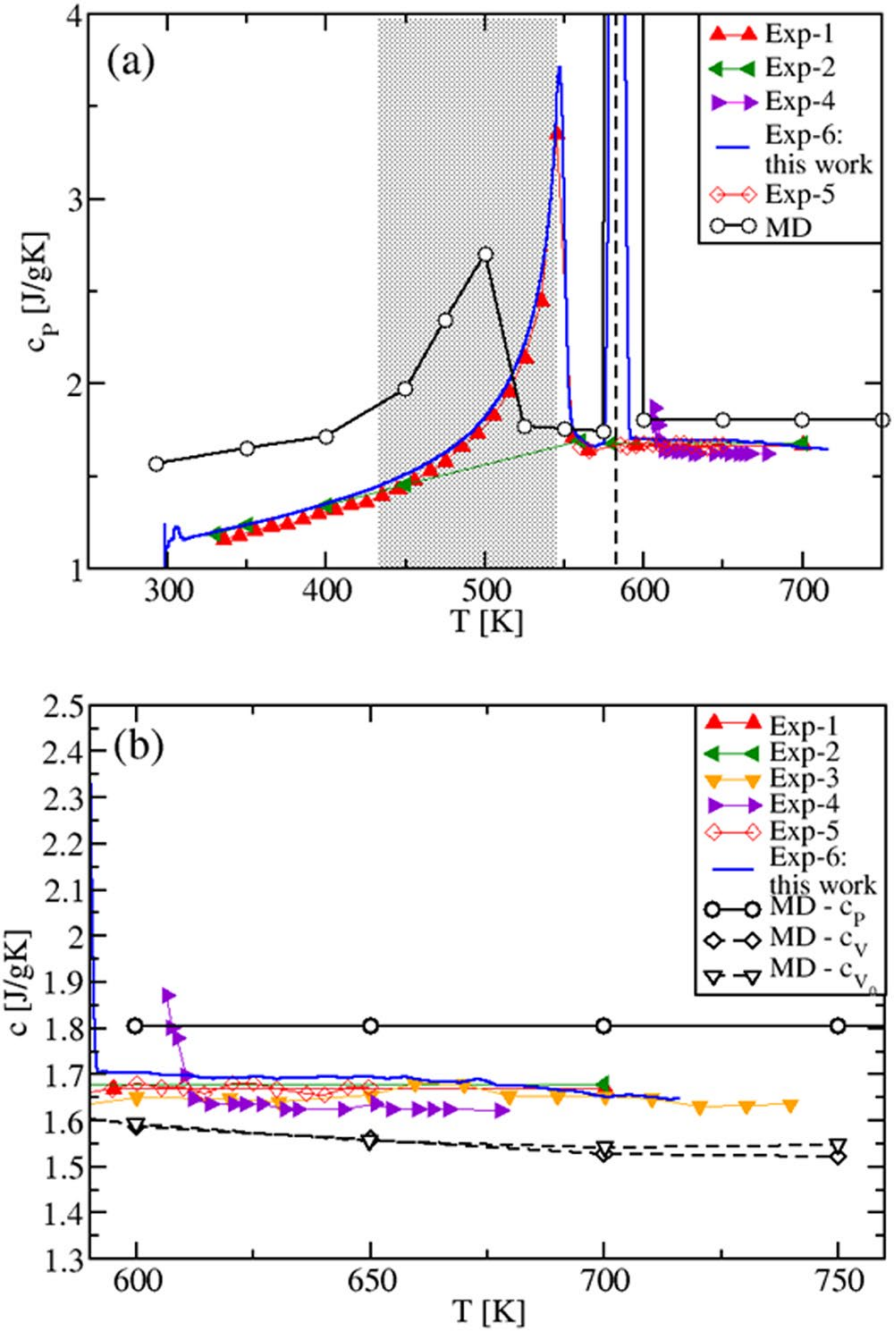
(**a**) Specific heat of NaNO_3_ in the whole solid (at *P* = 1 atm) and liquid regime investigated. In the solid phase, *c*_*P*_ increases with *T* and shows a peak in the same region where the second-order phase transition II-NaNO_3_ to I-NaNO_3_ is experimentally observed. The dashed vertical line corresponds to the experimental melting transition. (**b**) Specific heats *c*_*P*_ and *c*_*V*_ of NaNO_3_ in the liquid *T*-range only. As in KNO_3_, the simulated *c*_*P*_ is found to be constant, while *c*_*V*_ and ${c}_{{V}_{0}}$ are decreasing with temperature. Exp-1: ref.^4^; Exp-2: ref.^7^; Exp-3: ref.^6^; Exp-4: ref.^52^; Exp-5: ref.^53^.

The *c*_*P*_ values are shown in Fig. 6. In the solid phase, *c*_*P*_ increases with *T*, and shows a peak in the same region where the second-order phase transition is experimentally observed^26,34^. This transition is also evident from the *c*_*P*_(*T*) increase observed in our DSC experimental data, also reported in the same Figure. In the liquid phase, *c*_*P*_(*T*), analogously to KNO_3_, has a *constant value* for all the investigated temperatures (${c}_{P}^{MD}=1.805$ Jg^−1^ K^−1^), as shown in Fig. 6(a,b). Experimental *c*_*P*_(*T*) data are either constant, as the MD results, or exhibit a decreasing behavior. The comparison between our experimental and theoretical results show a difference of ≈7%. Instead, our MD *c*_*V*_(*T*) and ${c}_{{V}_{0}}(T)$ show a decreasing behavior.

#### Isothermal compressibility

**Figure 7:**
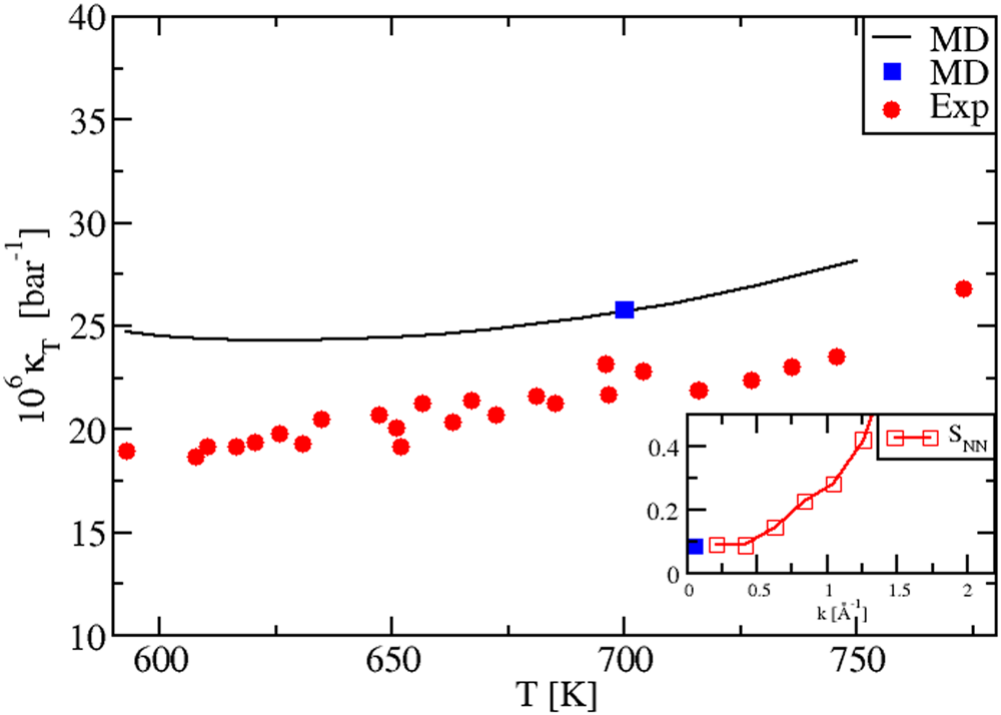
**(a)** Isothermal compressibility *κ*_*T*_(*T*) of NaNO_3_, calculated with three different methodologies, as in KNO_3_ (see Fig. 4 caption). Exp: ref.^50^. The black plot is calculated from the relation *κ*_*T*_ = *α*^2^*T*/[*ρ*(*c*_*P*_ − *c*_*V*_)]. The blue dot at *T* = 700 K is computed via an independent procedure and using *κ*_*T*_ = −*V*^−1^(∂ 〈*V*〉/∂*P*)_*T*_. As in KNO_3_, the blue dot is on top of the first plot and the Bhatia-Thornton structure factors shown in the inset tend to *κ*_*T*_ in the *k* → 0 limit.

Finally, as shown in Fig. 7, the *T*-behavior of the experimental isothermal compressibility *κ*_*T*_(*T*) in the liquid regime is also well reproduced by our MD modeling. A similar self-consistency test as for KNO_3_, based on the use of three calculation procedures was performed for NaNO_3_. The test was successful, as shown by the coincidence of the black plot and the blue dot in Fig. 7 and by the correct behavior of the Bhatia-Thornton structure factors *S*_*NN*_(*k*), tending to *κ*_*T*_ at the *k* → 0 limit. Hence, all the considerations on accuracy and precision made for the KNO_3_ isothermal compressibility apply here too.

### Eutectic and “Solar” Mixtures

Molten salt mixtures are technologically extremely relevant, especially when considered in their liquid phase. The characterization of their thermostatic properties is important not only for a fundamental understanding of the physics of ionic liquids at high temperature, but also for energy applications in any system containing a heat storage component. In this respect, it is also important the development of viable simulation methods to determine technological relevant binary and ternary molten salt eutectics^37^. In the following we will calculate, in the liquid phase only, the thermostatic properties of two relevant nitrate molten salt mixed systems: the *eutectic* and the *“solar”* mixture. Emphasis is given on the specific heats thermal behavior, where the available experiments still exhibit a high degree of controversy^4–7^.

The eutectic NaNO_3_-KNO_3_ mixture has the chemical composition Na_0.5_K_0.5_NO_3_. Due to the lower mass of Na, this corresponds to a 45.67% NaNO_3_–54.33% KNO_3_ weight percentage composition. The “solar salt” mixture has a higher content of Na, with a chemical composition of Na_0.641_K_0.359_NO_3_ and a weight percentage of 60% NaNO_3_–40% KNO_3_.

#### Mass densities

**Figure 8:**
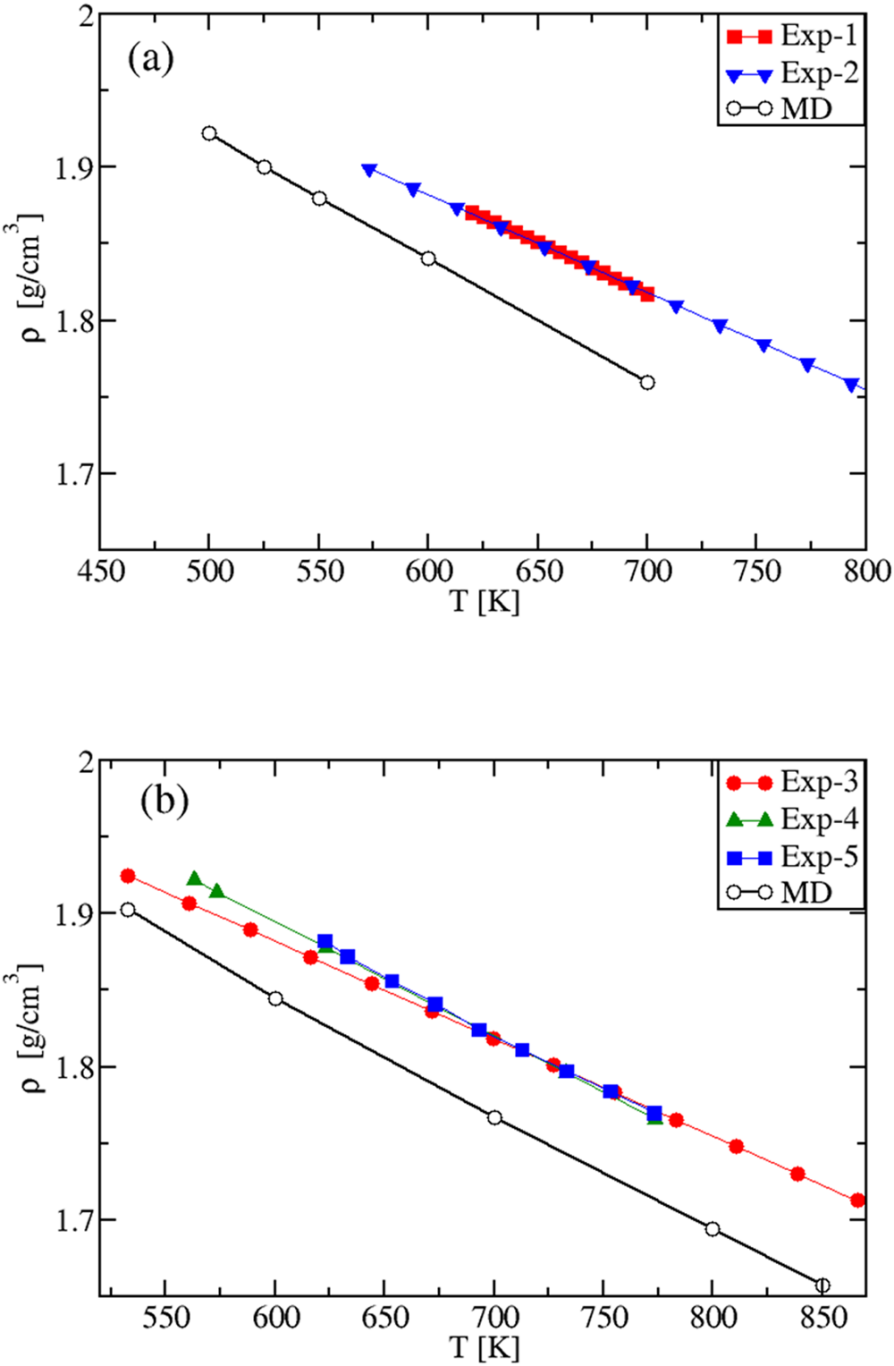
(**a**) Density *ρ*(*T*) of (**a**) the eutectic mixture and (**b**) the “solar” mixture in the liquid phase; Exp-1: ref.^54^; Exp-2: ref.^55^; Exp-3: ref.^9^; Exp-4: ref.^56^; Exp-5: ref.^57^.

The density plots for both systems are shown in Fig. 8. The theory-experiment agreement is satisfactory. However, while for the pure salts the difference between experimental and modeling results is the same in the whole liquid phase, here the discrepancy increases as the temperature increases. This behavior could be related to the use of the Lorentz-– Berthelot approximation^38^ to describe the crossed interaction between Na^+^ and K^+^ particles, which is the only approximation introduced in passing from the pure components to the mixtures.

#### Specific heats

**Figure 9:**
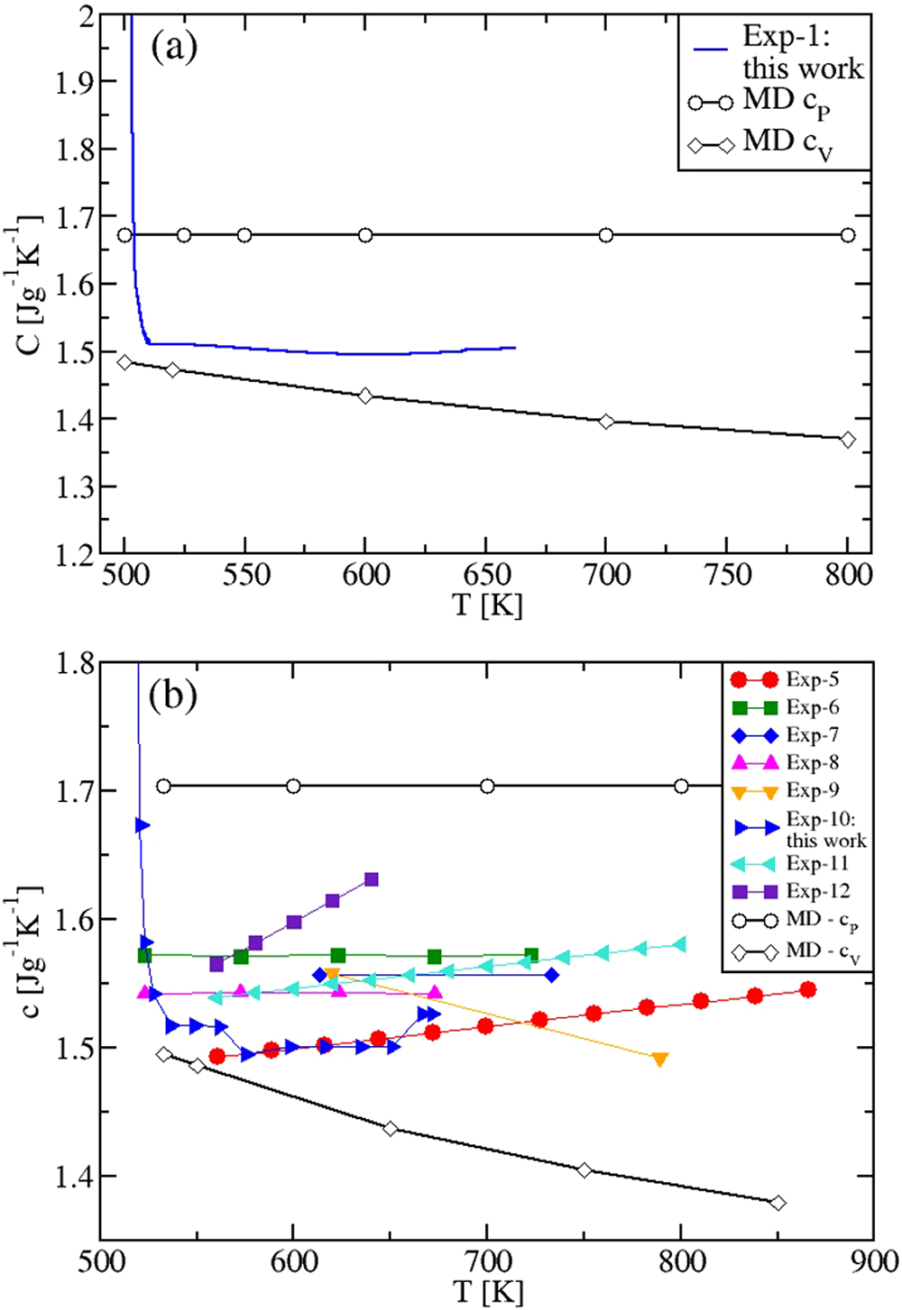
(**a**) Specific heats *c*_*P*_ and *c*_*V*_ of the eutectic mixture in the liquid phase; Exp-1: this work. (**b**) Same as (**a**) but for the “solar” mixture. In this latter case, the experimental picture is rather complex, with decreasing/constant/increasing/oscillating behaviors of the specific heat with the temperature. Our calculated value for *c*_*P*_ and *c*_*V*_ are constant and decreasing, respectively. This is similar to what found in the pure salts. Note, in particular, that our calculations revisit empirical relations used industrially^9^, showing an increasing *c*_*P*_ (turquoise left triangles). Exp-5: ref.^6^; Exp-6: ref.^7^; Exp-7: ref.^4^; Exp-8: ref.^5^; Exp-9: ref.^6^; Exp-10: this work.; Exp-11: ref.^9^; Exp-12: ref.^19^.

The specific heats of the mixtures are shown in Fig. 9(a,b). Importantly, we found again that the calculated values of *c*_*P*_ are *temperature independent*. They are 1.673 Jg ^−1^ K^−1^ and 1.704 Jg ^−1^ K^−1^ for the eutectic and the “solar” mixture, respectively. The specific heat *c*_*V*_(*T*), instead, is predicted to be a decreasing function of *T*. As the simulations allow to compute the specific heats by keeping either the volume or the pressure strictly constant, we speculate that all the observed systematic discrepancy between the *c*_*P*_(*T*) experimental and MD results can be due to the fact that in DSC experiments aimed at measuring *c*_*P*_(*T*), the control of the experimental conditions is rather challenging, especially in the case of ionic liquids. For instance, inside the sealed sample holders partially filled by a small amount of sample, a very high and variable pressure can be generated, with the sample changing its volume as the temperature is changed. Ionic samples present extra difficulties due to the non-wetting of the sample holder surfaces. This complexity is reflected in the variety of *c*_*P*_(*T*) behaviors with temperature, as measured by DSC. As shown in Figs 3(b), 6(b) and 9(a,b), *c*_*P*_(*T*) is found to decrease, to be constant and to increase. Our MD results clarify, once for all, that working at constant pressure a *constant value* of *c*_*P*_(*T*) is produced, while at constant volume a decreasing *c*_*V*_(*T*) is predicted.

As a conclusion for technological applications, we then propose to reconsider the use of empirical equations showing a *c*_*P*_(*T*) temperature-dependent behavior for any molten K-Na nitrate mixture (see, e.g., the *increasing c*_*p*_(*T*) used industrially, Exp. 11^9^ in Fig. 9(b)). We estimate that such a behavior, bringing, e.g., to a 5% overestimate of the real *c*_*p*_(*T*) value, corresponds to a loss of 0.75 h/day of electricity production in a CSP plant with a “solar” salt tank able to store energy for 15 h. On a yearly basis (and for a typical 50 MW plant), this traslates in the considerable loss of ≈12 GWh/year in the electricity production.

**Figure 10:**
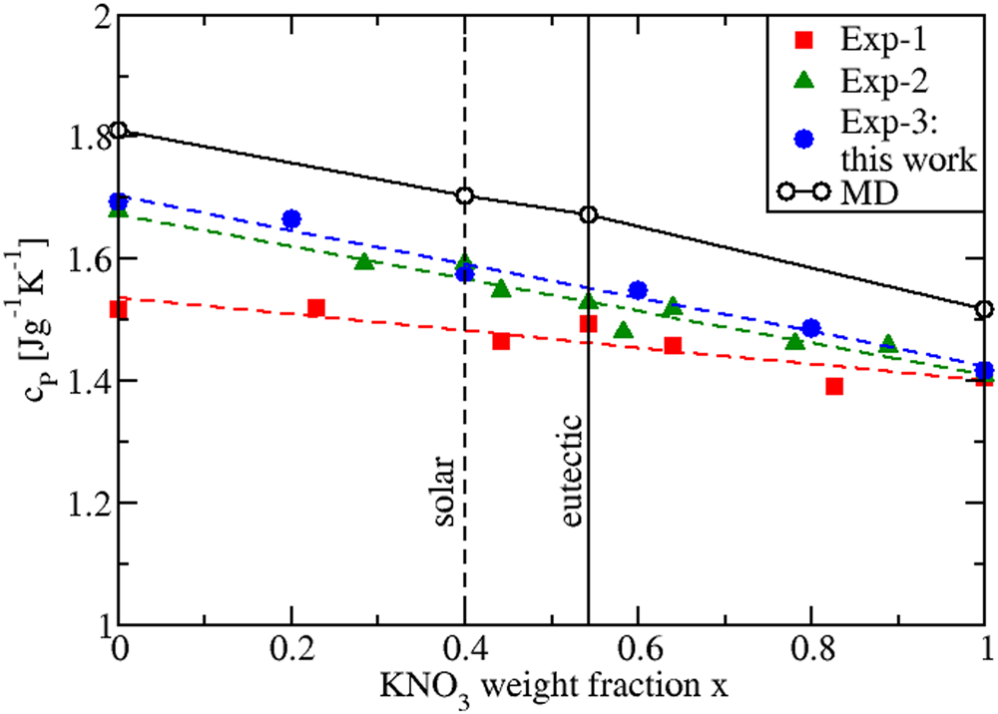
Specific heat *c*_*P*_(*x*) of as a function of the KNO_3_ weight fraction *x* (black plot). The vertical dashed line corresponds to the “solar” mixture, the plain to the eutectic. Dashed plots are linear interpolations of the experimental results; Exp-1: ref.^58^; Exp-2: ref.^7^; Exp-3: this work. Due to their *T*-dependence, all experimental values refer to averaged *T* values.

Due to the fact that the calculated *c*_*P*_ values of the pure and mixed salts are *T*-independent, *c*_*P*_ can be plotted against the weight fraction *x* of KNO_3_ present in the mixture. This is done in Fig. 10, where we see that by interpolating the calculated values of the four investigated compositions (pure NaNO_3_, eutectic, “solar salt” and pure KNO_3_), *c*_*P*_ changes linearly. Although the experimental data in Fig. 10 refer to different temperatures, a linear behavior can be also identified. We then conclude that mixtures of KNO_3_ and NaNO_3_ behave as ideal mixtures. Thus, to determine the *c*_*P*_(*x*) of *any* mixture, no extra measurements or simulations are needed, as it is sufficient to consider a linear combination of the pure salts values with their respective weight fractions, i.e. ${c}_{P}(x)=x{c}_{P}^{KN{O}_{3}}+\mathrm{(1}-x){c}_{P}^{NaN{O}_{3}}$.

#### Isothermal compressibility

**Figure 11:**
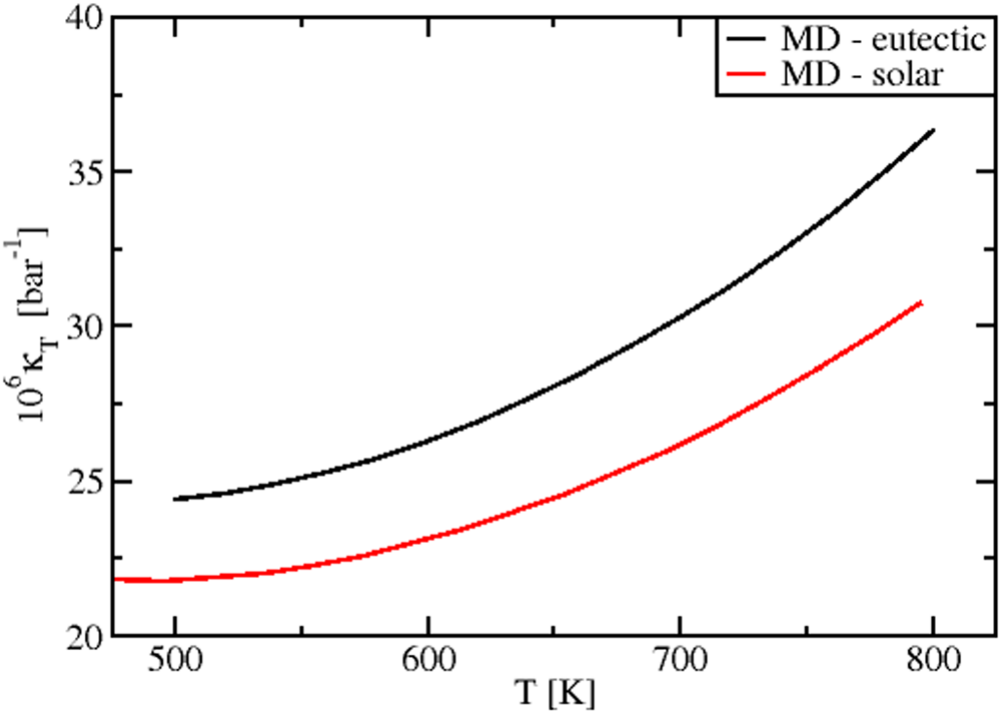
MD predicted isothermal compressibility, *κ*_*T*_(*T*), for the eutectic and “solar” mixtures.

The MD values of the isothermal compressibility *κ*_*T*_(*T*) are reported in Fig. 11. These data are the first prediction appearing in literature. By considering the accuracy of the MD results for the pure salts, highlighted in previous sections, they can be used as reference for further studies, both experimental and theoretical.

## Discussion

In this work, by combining classical molecular dynamics (MD) simulations and differential scanning calorimetry (DSC) experiments, we investigated the thermostatic properties of nitrate molten salts, technologically relevant materials for thermal energy storage applications. We focussed, in particular, on the thermal behavior of the specific heats of KNO_3_, NaNO_3_ and their eutectic and “solar” mixture, the latter known as “solar salt”. The motivation of our work is twofold: First, to the best of our knowledge, theoretical calculations about the thermostatic properties in the solid and liquid phase of these materials are largely missing. Second, there is a general lack of consensus about the experimentally measured specific heats as a function of temperature, especially for what concerns *c*_*P*_ in the liquid phases.

To address these issues, we first computed the mass density, the enthalpy and the thermal expansion coefficient of pure nitrate salts, as a function of temperature. Moreover, we calculated the melting transition temperature *T*_*M*_ and the enthalpy and the volume changes at *T*_*M*_. We obtained an accurate theoretical description, reproducing quantitatively the available experimental data. This allowed to reproduce several non-trivial features of solid-solid and solid-liquid phase transitions in the case of the pure salts.

Next, we characterized the specific heats in the pure salts, and, due to their technological relevance, in the mixtures. For all investigated liquid systems, we found a *constant* value of *c*_*P*_(*T*), while *c*_*V*_(*T*) is weakly decreasing with temperature.

Finally, we calculated the isothermal compressibility *κ*_*T*_(*T*), encompassing other thermostatic properties, in excellent agreement with the experiment. This fact, together with a careful, self-consistency check based on *three* independent procedures to calculate this quantity, fully validates our MD numerical scheme. Thus, we expect that our results for the eutectic and “solar” mixtures have the same degree of accuracy.

The constant value of *c*_*P*_(*T*) in the liquid regime clarifies a complex experimental picture, especially in the case of the “solar” mixture, where more experimental data are available. In view of this, for this material we suggest to reconsider the empirical *T*-increasing function *c*_*P*_(*T*) = *γ* + *δ T*, used in the design of thermal energy storage components.

For the eutectic and “solar” salt our results also allow to say that these materials behave as ideal mixtures, i.e. the *c*_*P*_ of *any* mixture can be obtained from the *c*_*P*_ of the pure salts only.

We believe that our results are of general validity and not limited to the class of nitrate molten salts. They confirm many temperature trends observed in the thermostatic properties of strongly interacting liquids (e.g the condensed phases of alkali halides^31^). They provide guidelines for researchers who perform experiments on the development of salt-based fluids for thermal energy storage. These include bulk nanomaterials and colloidal suspensions in ionic compounds.

Future studies will require theoretical reference data and validated models about basic materials that compose more complex nanomaterials, as the ones provided in this work.

## Methods

### Computational Methods

The used classical MD model is based on a version of the Fumi and Tosi pair interaction potential^39,40^, i.e. the Buckingham potential, superimposed to a Coulomb potential. The inter-atomic parameters of the Buckingham potentials are taken from S. Jayaraman *et al*.^13^. This parametrization has been chosen because: (i) it reproduces the liquid and crystal phase densities of the pure KNO_3_ and NaNO_3_ within 4% of the experimental data^13^; (ii) it reproduces the MD partial pair distribution functions, *g*_*ij*_(*r*), of the pure NaNO_3_ evaluated by A.K. Adya *et al*.^41^ with a Born-Mayer-Huggins type of interaction potential. The latter reproduces the experimental structure factor extracted from the measured X-ray diffraction intensity^41^. No further refinements of the interaction parameters, and no other approximations beyond the Lorentz– Berthelot approximation^38^, needed for the cross interaction parameters in the mixture case, are made. The model is sufficiently accurate for the purposes of the present study. Simulations have been performed with the LAMMPS code^42,43^. Initial solid configurations are taken from ref.^13^. The NaNO_3_ initial solid configuration at *T* = 293 K corresponds to the rhombohedral $R\bar{{\rm{3}}}c$ group with Z = 6, **a** = 5.070 *Å*, **c** = 16.82 *Å*. The KNO_3_ initial solid configuration at T = 293 K corresponds to the orthorhombic Cmc2 group with Z = 16, **a** = 10.825 *Å*, **b** = 18.351 *Å*, **c** = 6.435 *Å*. The initial liquid configurations have been generated with the PACKMOL package^44^. To study the pure salts, systems with 600 cations and 600 ${{\rm{NO}}}_{3}^{-}$ ions, for a total of 3000 particles, were constructed. To study the mixtures, 600 ${{\rm{NO}}}_{3}^{-}$ ions have been used while the cation numbers were 300 Na^+^ and 300 K^+^ for the equimolar mixture, and 384 Na^+^ and 216 K^+^ for the solar mixture. A time-step of 1 fs has been used. Systems were equilibrated using 10^6^ time-steps and run with further 10^6^ − 2 × 10^6^ time-steps. For the solid (liquid) systems, equilibrated configurations at the lowest (highest) studied temperature were used as input for the closest next higher (lower) temperature. Other relevant parameters used are: Nosé-Hoover barostat time constant 0.5 ps; Nosé-Hoover thermostat time constant 0.1 ps; Buckingham and Coulomb interaction cutoff distance *r*_*c*_ = 12 *Å* for systems with K^+^ ions, and *r*_*c*_ = 11 *Å* for other cases; long-range force calculation accuracy 10^−4^. Production runs in NPT simulations were considered suitable for analysis only when the averaged equilibrated pressure was within 〈*P*〉 = 1 ± 1 atm. Within this range, the change in the averaged 〈*H*〉 is $\le 1\times {10}^{-2} \%$.

### Thermodynamic Properties

Thermodynamically, the specific heats *c*_*V*_ and *c*_*P*_ are defined as:

1 $${c}_{V}=\frac{1}{N}{(\frac{\partial E}{\partial T})}_{V,N},$$

2 $${c}_{P}=\frac{1}{N}{(\frac{\partial H}{\partial T})}_{P,N},$$

where *E* is the internal energy, *H* the enthalpy and *N* the mole number, which is kept constant. These quantities are linked by the relation:

3 $${c}_{P}={c}_{V}+\frac{1}{\rho }\frac{{\alpha }^{2}}{{\kappa }_{T}}T,$$

where *α* = *V*^−1^(∂*V*/∂*T*)_*P*_ is the coefficient of thermal expansion, *ρ* = *N*/*V* is the density and *κ*_*T*_ = −*V*^−1^(∂*V*/∂*P*)_*T*_ the isothermal compressibility. Eq. (3) can be also written in the form:

4 $${c}_{P}={c}_{V}\mathrm{(1}+\gamma \alpha T),$$

were the Grüneisen parameter, *γ* = *α*/(*ρκ*_*T*_*c*_*V*_), is introduced. Typically, *γ* ≈ 1 and it is almost T-independent^30^.

In the *NVT* ensemble, the internal energy *E* in Eq. (1) is expressed by the relation:

5 $$E={E}_{Kin}(T)+{E}_{Pot}(T)={E}_{Kin}(T)+2\pi \rho TN\sum _{i,j}{x}_{i}{x}_{j}\int {E}_{ij}^{pot}(r){g}_{ij}(T,r){r}^{2}{\rm{d}}r\,,$$

where *x* indicates the number fraction and *g*_*ij*_(*T*, *r*) the *ij* pair distribution function. If in the considered T-range all the kinetic degrees of freedom are activated, *E*_*Kin*_ is a linear function of *T* giving a constant term contribution to *c*_*V*_(*T*). Then the temperature contribution to *c*_*V*_(*T*) only arises from the *T*-dependence of the density *ρ* and of the pair distribution functions *g*_*ij*_(*r*) appearing in the *E*_*Pot*_ term. To separate the temperature effects of these two quantities, two sets of simulations have been conducted. In the first set, a liquid density *ρ*_0_, chosen at a density close to the freezing point, is kept fixed for all the investigated *T*; in the second set, the *ρ*(*T*) values obtained from the *NPT* equilibration run are used.

### Simulation Methods and Self-Consistent Check

To calculate the specific heats and other thermostatic properties in the liquid phase, the simulation is performed by a step-by-step cooling procedure. First, we worked in the *NPT* ensemble. Starting from a random distribution of particles^44^, the system is equilibrated at the highest temperature, usually the experimental decomposition temperature where the salt molecules break. The temperature is then decreased, and the equilibrated configuration of the previous *T*-step is used as a starting configuration for the next *T*. All equilibrated configurations are used to perform production runs and data analysis. When necessary, these *NPT* configurations are used as input for *NVT* ensemble simulations.

As for the specific heats, there are several ways to determine them from ensemble averages of MD trajectories^38^. In this work, the direct evaluation via the relations

6 $${c}_{V}={(\frac{\partial {\langle E\rangle }_{NVT}}{\partial T})}_{N,V},\,\,\,{c}_{P}={(\frac{\partial {\langle H\rangle }_{NPT}}{\partial T})}_{N,P},$$

is preferred due to its stability and reliability. Their evaluation is functional to a scheme aimed at the determination of all thermostatic properties. Moreover, a final self-consistency check is performed.

In detail:

1. Fix the pressure, *P* = 1 atm, and the number of moles *N*.
2. Perform *NPT* simulations for a set of temperatures *T*.
3. Extract the functions: *V*(*T*), *ρ*(*T*), *α*(*T*) = *V*^−1^(∂ 〈*V*〉/∂*T*)_*P*_, *H*(*T*) and *c*_*P*_(*T*) from Eq. (6).
4. For each value *V*(*T*_*i*_) perform four *NVT* simulations at temperatures *T*_*i*_ ± Δ*T* and *T*_*i*_ ± 2Δ*T*.
5. Extract *c*_*V*_(*T*_*i*_) from Eq. (6), via a four-point differentiation relation of the total energy *E*(*T*)^45^.
6. Repeat the previous point for a set of temperatures and obtain the function *c*_*V*_(*T*).
7. Calculate the isothermal compressibility *κ*_*T*_, via *three* different procedures. *First*, for each *T*_*i*_ perform a set of *NPT* simulations in the pressure interval *P* ∈ [1, 600] atm. Use the equation
  7 $${\kappa }_{T}=-\,{V}^{-1}{(\partial \langle V\rangle /\partial P)}_{{T}_{i}},$$
  to extract *κ*_*T*_(*T*_*i*_) from a linear differentiation.
8. Repeat the previous point for a set of temperatures and obtain the function *κ*_*T*_(*T*).
9. Self-consistency check:
  - Compare the *κ*_*T*_(*T*) values from step 8 with a *second* set of values, from the equation
    8 $${\kappa }_{T}={\alpha }^{2}T/[\rho ({c}_{P}-{c}_{V})]$$
    where *α*(*T*), *ρ*(*T*) and *c*_*P*_(*T*) are obtained from step 3, and *c*_*V*_(*T*) from step 6.
  - Evaluate the percentage difference of *κ*_*T*_ calculated in the two ways. This difference is a severe test for: (i) the achievement of equilibrated MD configurations (slope of the equilibrated energy functions 〈10^−8^ ÷ 10^−9^); (ii) the numerical precision of the procedures to determine *c*_*P*_, *c*_*V*_, *α*, and *κ*_*T*_ itself.
  - *κ*_*T*_ can also be determined via a *third* procedure, from the total number density Bhatia-Thornton structure factor, *S*_*NN*_(*k*)^25,32,33^:

9 $${\kappa }_{T}=\frac{1}{{\rho }_{N}{k}_{B}T}\mathop{\mathrm{lim}}\limits_{k\to 0}{S}_{NN}(k),$$

where

10 $${S}_{NN}(k)=1+\rho [{x}_{c}^{2}{\tilde{h}}_{cc}(k)+{x}_{a}^{2}{\tilde{h}}_{aa}(k)+2{x}_{c}{x}_{a}{\tilde{h}}_{ca}(k)],$$

In these equations, *ρ*_*N*_ is the number density and ${\tilde{h}}_{\alpha \beta }(k)$ are Fourier transform of the cation and anion pair correlation functions *h*_*αβ*_(*r*) = *g*_*αβ*_(*r*) − 1. Here, the pair distribution functions *g*_*αβ*_(*r*) are obtained from the *NVT* averages ${x}_{\alpha }{x}_{\beta }\rho {g}_{\alpha \beta }(r)={N}^{-1}\langle {\sum }_{{i}_{\alpha }}{\sum }_{{j}_{\beta }}\delta (r+{r}_{{i}_{\alpha }}-{r}_{{j}_{\beta }})\rangle$. This calculation of *κ*_*T*_ through microscopic structural quantities, is a further check of the overall numerical scheme precision.

Calculations in the solid phase follow the same lines sketched for the liquid phase. The only difference is in the sequence of the MD runs, as in this case, in general, the simulations at constant pressure were performed as a step-by-step *heating* procedure. As for KNO_3_, we additionally adopted a step-by-step *cooling* procedure. In the initial configuration at the lowest temperature (*T* = 300 K), we considered the energetically stable crystallographic configuration at that temperature^13,26^.

### Method to Simulate the Melting Transition Temperature

**Figure 12:**
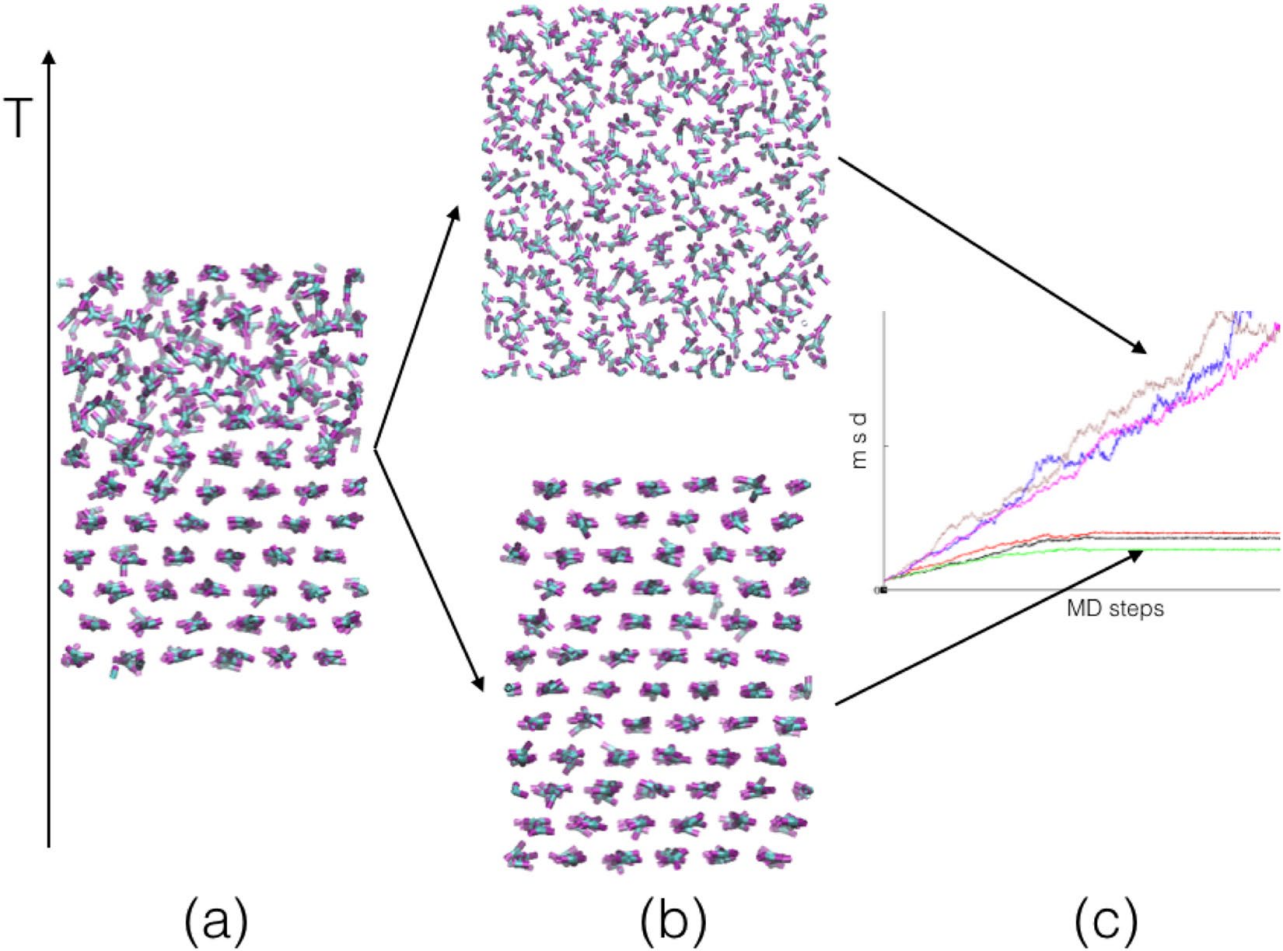
Procedure to calculate the solid-to-liquid transition temperature *T*_*M*_ by simulating the temporal evolution of a two-phase system. (**a**) System where the two phases coexist. (**b**) Depending whether the simulation temperature is ${T}_{S} > {T}_{M}$ or ${T}_{L} > {T}_{M}$, the system will evolve, respectively, either towards a solid phase (lower configuration) or a liquid phase (upper configuration). **(c**) Mean squared displacement (*msd*) as a function of the MD time steps: a zero, constant or changing slope indicates, respectively, a solid phase, a liquid phase and an evolving phase towards one of them.

To calculate the solid-to-liquid transition temperature *T*_*M*_, we adopted an approach based on the direct simulation of a two-phases coexistence, with an explicit interface (Fig. 12). This approach has proven to be robust and reliable for systems of large size in particle number^46–48^. Figure 12(a) shows a system with solid and liquid phases, previously (and separately) equilibrated at a temperature close to the expected *T*_*M*_. By performing a simulation at ${T}_{L} > {T}_{M}$, the interface will move in order to suppress the solid phase (Fig. 12(b), upper panel), while at *T*_*S*_ < *T*_*M*_, the interface will move in order to suppress the liquid phase (Fig. 12(b), lower panel). The evolution of the two-phase system towards either the liquid or the solid phase is shown in Fig. 12(c). Here the mean squared displacement (*msd*) of the atoms is plotted as a function of the MD time steps. The solid will be characterized by a zero slope and the liquid by a slope related to the liquid self-diffusion constant. The change in slope for increasing time steps will characterize the two-to-one phase transition, in both cases. After having chosen a suitable temperature interval (which must include *T*_*M*_), the procedure to obtain the transition temperature consists in starting from the configuration in Fig. 12(a) and performing a set of simulations by slowly decreasing (increasing) *T*_*L*_ (*T*_*S*_) until the difference Δ*T* = *T*_*L*_ − *T*_*S*_ is small enough. In our simulations we chose Δ*T* ≈ 5 K, as smaller values make our results unstable and strongly dependent on the coupling rates with the thermostat and the barostat. This small value defines the accuracy of the calculated melting temperature, *T*_*M*_ = (*T*_*L*_ + *T*_*S*_)/2. This approach will also allow to calculate the volume and enthalpy changes at the transition, respectively Δ*V*_*M*_ = *V*_*L*_(*T*_*M*_ + Δ*T*/2) − *V*_*S*_(*T*_*M*_ − Δ*T*/2) and Δ*H*_*M*_ = *H*_*L*_(*T*_*M*_ + Δ*T*/2) − *H*_*S*_(*T*_*M*_ − Δ*T*/2).

### Experimental

For the four investigated systems, we have performed a set of differential scanning calorimetry measurements (DSC). High purity sodium nitrate and potassium nitrate were provided by Sigma Aldrich and the salts were used without any further purification for the present study. In order to remove the final traces of moisture, the samples were heated at 293 K with a heating plate located inside a globe box, and then the samples were encapsulated using hermetic Tzero aluminium lids and pans at argon atmosphere. Pure salt, standard sapphire and a reference (only the Al crucible) were hermetically sealed at argon atmosphere. The sapphire and the samples weights were measured by using a microbalance to 4 decimals in milligram (Mettles Toledo. X6TU Model). The specific heat values of all the samples were measured by using modulated differential scanning calorimeter (MDSC) (TA instruments, Q2000) with specific heating program. The encapsulated samples were heated at 723 K and kept isothermal for 10 minutes (to stabilize the heat flux signal). Before that, Tzero heat flow was implemented at this temperature and the sample was equilibrated at 298 K and then ±1 K was executed every 120 seconds and stay again isothermal for 10 minutes. Finally, 2 K/min heat ramp was implemented till reach at 723 K.

### Data availability

The datasets generated during and/or analyzed during the current study are available from the corresponding author on reasonable request.
